# Education, Training, and Practices of Neurorehabilitation in India During the COVID-19 Pandemic

**DOI:** 10.3389/fneur.2021.626399

**Published:** 2021-02-10

**Authors:** Nirmal Surya, Abhishek Srivastava, Taral Nagda, Deepak Palande, Hitav Someshwar

**Affiliations:** ^1^Epilepsy Foundation India, Mumbai, India; ^2^Bombay Hospital, Mumbai, India; ^3^Kokilaben Dhirubhai Ambani Hospital and Medical Research Institute, Mumbai, India; ^4^Society for Rehabilitation and Care of Children Hospital, Mumbai, India; ^5^Grant Medical College and Sir Jamshedjee Jeejeebhoy Group of Hospitals, Mumbai, India; ^6^KJ Somaiya College of Physiotherapy, Mumbai, India

**Keywords:** neurorehabiliation, COVID-19, India, pandemic, education

## Abstract

**Background:** Corona virus disease (COVID-19) was declared a pandemic by the World Health Organization in March 2020. This has affected service delivery among all medical disciplines in India including neurorehabilitation services.

**Aims and Objectives:** The aims and objectives of the study were to assess the effect of COVID-19 pandemic on neurorehabilitation services across India.

**Methodology:** A prospective nationwide survey study was undertaken by the Indian Federation of Neurorehabilitation during the pandemic. A questionnaire was prepared using Google forms software consisting of four sections: demography, neurorehabilitation practice before COVID-19 pandemic, neurorehabilitation practice during COVID-19 pandemic, and continuing medical education during COVID-19 pandemic.

**Results:** Responses (872) were received from neurorehabilitation professionals across the country out of which 2.2% professionals did not give consent for participating in the survey. Participants (36.6%) were practicing traditional or independent referral basis rehabilitation, while 63.4% participants were practicing multidisciplinary rehabilitation. On an average, respective units were conducting 500–750 therapy sessions per month. Majority of the rehabilitation units in India lacked a physiatrist, rehabilitation nurse, music therapist, cognitive therapist, and urologist. Approximately 80% of the rehabilitation units have the basic rehabilitation modalities and advance technology was present in only 20% of the rehabilitation units. During COVID-19 pandemic, 19.5% centers were providing elective services, 50.3% emergency services, 15.6% new outpatient services, and 22.7% were providing follow-up outpatient services. Centers (51.5%) were providing telerehabilitation services for neurological conditions during the times of COVID-19 pandemic. Professionals (61.1%) providing telerehabilitation were working from home. Among the patients who needed neurorehabilitation, 28% were doing their exercises independently, 31% were supervised by caregivers, 17% were supervised by therapists, and 24% were not receiving any therapy. Participants (95.5%) wanted to receive more training in the field of neurorehabilitation. The participants utilized webinars (71%), online courses (22%), case discussion forums (19%), panel discussions (13%), and literature search (8%) during COVID-19 pandemic to continue education.

**Conclusion:** The study reflects the situation of neurorehabilitation service delivery in India during the pandemic as the respondents were from all parts of the country and included most components of the neurorehabilitation team. Neurorehabilitation services were severely affected across India during the COVID-19 pandemic. Tele-neurorehabilitation has emerged as a new service delivery model during the pandemic. Online means of education has emerged as the primary source of continuing medical education during the pandemic.

## Introduction

A new type of respiratory disorder was reported in Wuhan, China, which was identified as a novel virus on December 31, 2019. The World Health Organization called it the novel COVID-19 virus on February 11, 2020. Coronavirus also known as COVID-19 belongs to a group of pathogens that target the pulmonary system in humans. They are primarily non-segmented positive sense RNA viruses (1). The World Health Organization declared it as a pandemic on March 11, 2020. India received its first case of the COVID-19 pandemic on January 30 in the state of Kerala; the patient had a positive history of travel to Wuhan, China. As of August 22, 2020, 23,121,145 people in 213 countries and two international conveyances have been infected by the COVID-19 virus. In India, the situation is unpleasant, with an estimated population of 1.3 billion, the total number of COVID-19 positive cases are 2,975,000 which is the 3rd highest number of cases trailing behind Brazil and the United States (2).

During the early stage of the spread of the virus in India, there was not much burden on the chronic health care settings and outpatients departments, but as the situation escalated, a nationwide lockdown was enforced, and the movement of the common people was reduced to only for essentials. Many sectors in India have been affected due to the COVID-19 pandemic. The primary and acute services and gradually health care sector were over burdened with exponential increase in the number of cases. The neurorehabilitation sector was also affected due to these changing trends. Critical patients with neurological complications were shifted from intensive care units to inpatient wards as more and more beds were needed in the critical care units. Many outpatient departments stopped functioning or those which functioned were working at 25% capacity due to the restrictions imposed by the local governing bodies. This had a significant impact on the patients undergoing neurorehabilitation as their functional recovery was hampered due to the non-availability of rehabilitation services.

Since there was no data available on practice of neurorehabilitation services, neurorehabilitation training and education in India and developing countries during the COVID-19 pandemic, the Indian Federation of Neurorehabilitation conducted this study to determine the effects of COVID-19 pandemic on neurorehabilitation services across India, to determine the measures taken by the rehabilitation professionals and institutes providing neurorehabilitation during the pandemic, and also, to assess the effect of pandemic on education and training, and the role of e-learning.

## Methodology

We conducted a descriptive, cross sectional nationwide survey among neurorehabilitation professionals in India during the period of corona virus (COVID-19) outbreak from April–May 2020. The structure, need and the purpose of the study were explained to the participants, and the point that participation is voluntary was explained before taking the consent. All participants were included in the study only after they provided their written informed consent. The responses of the participants were kept anonymous.

An electronic questionnaire for the survey was developed by a group of experts consisting of neurologist, rehabilitation physician, pediatric, orthopedic surgeons, neurosurgeon, and physiotherapist (Supplementary Material). The electronic version of the questionnaire consisted of four sections:

1. Demographics
2. Neurorehabilitation practice before COVID-19 pandemic
3. Neurorehabilitation practice during COVID-19 pandemic
4. Continuing medical education during COVID-19 pandemic

The questionnaire consisted of 35 questions which included both open ended and close ended questions.

The questionnaire was scrutinized by two independent experts in the field of neurorehabilitation and research. The pilot study was conducted on 30 participants. Following the pilot study format of 12 questions was modified and two questions were clubbed. The changed questionnaire after the pilot was again face validated. The questionnaire was circulated to 3,368 neurorehabilitation professionals across the country via electronic mail. Only one response was accepted from each professional, and they were not allowed to change their answers once the response was submitted. The data was analyzed using descriptive statistics which included mean, standard deviation, frequency distribution, and percentages.

## Results

A total of 872 responses from neurorehabilitation professionals across the country were received. Professionals (853, 97.82%) gave electronic informed consent for participating in this survey.

### Demographics

**Figure 1:**
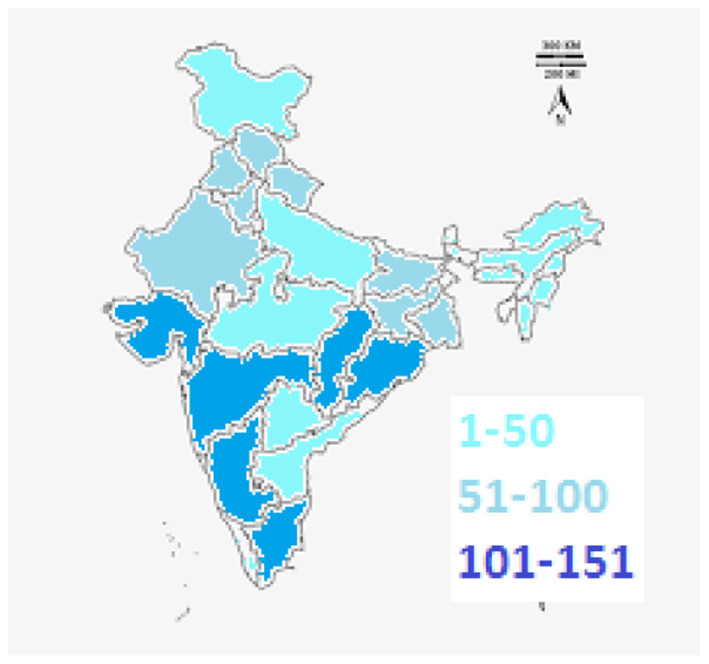
Map of India showing the number of responses from different states.

There were representations from all the states and union territories of India (Figure 1).

Table 1 shows the demographics of the study population in terms of area of practice, qualifications, experience in the field of neurorehabilitation, type of rehabilitation services offered, and the type of institute affiliation. Majority of the participants (727, 85.22%) were young rehabilitation professionals, practicing in urban setup (76.8%), and 658 (77.13%) participants were females and 195 (22.86%) participants were males. The participants were representative of all the professionals in a multidisciplinary team: physical therapy, 534 (62.6%), speech therapy, 112 (13.1%), occupational therapy, 101 (11.8%), physical medicine and rehabilitation, 45 (5.3%), neurology, 22 (2.6%), psychology, 8 (1%), orthopedics, 6 (0.7%), pediatrics, 3 (0.4%), and others, 22 (2.5%).

**Table 1:** Demographics of the study population.

**Age group**	**Number of participants**	**Percentage**
20–29 years	491	57.56%
30–39 years	236	27.66%
40–49 years	85	9.96%
50–59 years	24	2.81%
60 years and above	17	2.01%
Total	853	100%
**Qualifications**	**Number**	**Percentage**
Physiotherapist	534	62.6%
Speech and Language Pathologist	112	13.1%
Occupational Therapist	101	11.8%
Rehab Physician	45	5.3%
Neurologist	22	2.5%
Others	22	2.5%
Psychologist	8	1%
Orthopedic surgeon	6	0.7%
Pediatrician	3	0.4%
**Experience in the field of neurorehabilitation**	**Number**	**Frequency**
Undergraduate student	114	13.4%
Post Graduate student	155	18.2%
PhD student	13	1.6%
Fresher	71	8.1%
1–5 years	204	24%
6–10 years	127	14.9%
11–15 years	88	10.4%
>15 years	81	9.4%
**Type of rehabilitation services provided**		
Traditional	312	36.6%
Multidisciplinary	541	63.4%
**Type of institute affiliated**		
Teaching institute	357	41.9%
Private Hospital	156	18.3%
Government Hospital	68	7.9%
Specialized Rehabilitation Center	47	5.5%
Private Outpatient Clinic	181	21.2%
Others	44	5.2%

### Neurorehabilitation Practices Before the COVID−19 Pandemic

**Figure 2:**
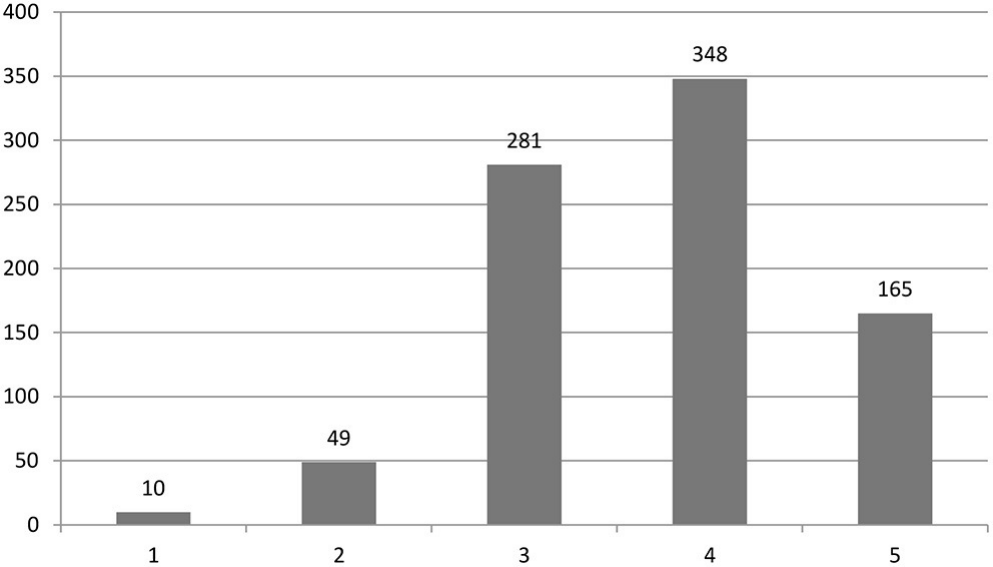
Self-rating of their respective rehabilitation units by the participants.

The most common diseases treated were: cerebral palsy (87.5%), Parkinson's disease (85.3%), traumatic brain injury (76.9%), migraine (32%), psychiatric disorders (30.2%), and chronic fatigue syndrome (20.2%). Figure 2 shows how the participants of the study rate their rehabilitation units on a scale of 1–5, with 1 being average and 5 being advanced. Majority of them rate their rehabilitation units 4 (40.8%) and 3 (32.9%) on a scale of 1–5.

The participants reported that on an average, their respective rehabilitation units were conducting ~500–750 therapy sessions per month. When asked regarding the composition of their respective multidisciplinary rehabilitation teams, majority of the neurorehabilitation teams in India did not have a physiatrist, rehabilitation nurse, music therapist, cognitive therapist, and urologist. Participants (706, 80.96%) reported that their neurorehabilitation units had basic rehabilitation modalities such as tilt board, parallel bars, mirror therapy, etc. Only 20% of the participants worked in neurorehabilitation units which had advanced rehabilitation modalities such as rehabilitation robotics, virtual reality training, and functional electrical stimulation, etc.

### Neurorehabilitation Practices During the COVID-19 Pandemic

As reported by the participants practicing in the urban set up, 680 (77.98%) participants were providing only emergency services, 732 (83.94%) were providing neurorehabilitation using teleconsultation and telerehabilitation; 170 (19.49%) were providing elective services, and only 70 (8%) were able to provide outpatient rehabilitation services. Majority of the rural set ups, 438 (50.3%) were providing outpatient services for new patients, and 532 (61.1%) were providing follow up services during the pandemic. Table 2 shows how often the neurorehabilitation professionals were called for duty during the pandemic times.

**Table 2:** Duty schedule for rehabilitation professionals during the pandemic times.

	**Number**	**Frequency**
Daily	187	21.9%
Three times a week	66	7.7%
Two times a week	42	4.9%
Once a week	37	4.3%
Work from Home	521	61.1%

### Effect of COVID-19 Pandemic for People With Disabilities

Out of the total patients requiring rehabilitation services during the pandemic at the units where the participants practice, 34% of their patients were receiving rehabilitation using telemedicine, 8% of their patients were admitted in the inpatient rehabilitation units, 32% of their patients were receiving rehabilitation by a trained family member or a caregiver for mobility, self-care, and communications needs, 17% of their patients were receiving home based or community based rehabilitation by a trained rehabilitation professional, and 9% of their patients were not receiving any form of neurorehabilitation care.

When we asked the participants “What are the three worst effects of COVID-19 pandemic for people with disabilities?” Participants (505, 57.91%) responded to the question. The responses are tabulated in Table 3.

**Table 3:** The effects of COVID 19 pandemic on people with disabilities.

**Psychological effects**	**Physical effects**	**Social effects**	**Effects on rehabilitation**
• Feeling of loneliness, helpless and anxious • Depression • Dependence • Morbidity mortality • Loss of confidence • Anxiety • Fear of death • Regression in condition • Mental breakdown • No motivation to do the exercises • Frustration • Susceptible to abuse • Behavioral issues	• Respiratory parameters • Musculoskeletal insufficiency • Irregular rehab session • Stiffness • Contractures • Loss of the achieved mobility • Loss of functional gains during recovery and lack of comprehensive rehab services • Atrophy • Pressure sores	• Altered routine • Lack of peer contact, friends and family contact • Reduced mobility due to being home bound • Participation restrictions • Transportation and daily needs • Earning loss, unable to get required rehab and reaching out to society	• Not able to come to hospitals • Lack of therapy/rehab • Lack of medical personnel • Danger of relapse of conditions due to nonavailability of medical care • Inability to consult a doctor when a genuine need is there • Fatigue

Those professionals providing neurorehabilitation services by coming in direct contact with patients wore masks, gloves, personal protective equipment, and followed disinfection regimen as advised by the World Health Organization (3) and Ministry of Health and Family Welfare, Government of India (4). The participants also reported that proper development of telerehabilitation protocols, proper training in telerehabilitation, and incorporation of family in the rehabilitation process can play a major role in adapting to the situation, and also providing diligent neurorehabilitation services. Majority of the participants answered that assessment, participation restriction, nonavailability of medications, poor follow up, difficulty using telerehabilitation for old age patients, fear of COVID infection especially in patients with Parkinson's disease, and multiple sclerosis, were the worst effects of COVID 19 pandemic on patients with neurological disabilities.

### Continuing Medical Education During and After COVID 19 Pandemic

Participants (320, 36.8%) felt that the curriculum was not adequate in undergraduates and post graduate courses; 832 (95.5%) participants wanted to receive more training in the field of neurorehabilitation during the pandemic. The time available during pandemic was utilized to continue education by participating in webinars (71%), online courses (22%), case discussion forums (19%), panel discussions (13%), and literature search (8%). During COVID-19 pandemic, 40% of the participants attended <5 webinars, 46.5% attended 5–10 webinars, and 13.5% attended more than 10 webinars per week. Sixty-three percentage participants felt that there will be a shift toward online courses, and 41% felt that things will be the same as it was before the pandemic.

When we asked the participants “How will education change in the future after COVID-19?,” the total 56% number of responders answered the question. The responses were categorized as predictive, positive, and negative, and after combining repetitive responses together, are listed in Table 4.

**Table 4:** How will education change in the future after COVID-19?

**Predictions**	**Positive effects**	**Negative effects**
• The online training will be an integrated part of education • Totally changed Online • Will be more virtual and lots of discussions • Pattern of assessment of examination may also change • More online education • Will overcome the traditional method of learning • Everything will change not education • More usage of technology and less dependent on writing work • Technology will continue to play a key role in educating future generations • Education around the globe should become education about the globe. • Education will become more virtual and technology dependent. Interaction may be compromised up to some extent. • Preparedness for unforeseen circumstances and alternative ways to cope for lack of resources in times of health crises will be a part of the education system. • People since now learnt the proper educational use of online classes it might be an evolution in education system. • It will be more of web and VR based. Physical meetings will be less frequent	• With social awareness more accessibility through online mode. • Will improve access to international standards • Enough time to learn new things, and relearn the subjects • More holistic approach. • It would be better as it is getting global • Many researchers would be enlightened with new ideas, and that can bring huge change after lockdown! • Education to go more in depth • Connecting more fellows, students, and researchers virtually and involving them in E learning, e rehab • Use of technology and more visual aids will facilitate better understanding • Reaching out to rural areas will now gain widespread acceptance. • Education always brings change in your way of thinking provided one uses education more effectively and for the benefit of the world. • Use of technology and more visual aids will facilitate better understanding. • Reaching out to rural areas will now gain widespread acceptance. • Now we can attend lectures by sitting in any part of the world and upgrade our knowledge. • More will be ready for online courses and study for which people were not ready earlier specially in India • Now people are free to read with no chaos of environment, no pollution, no exam or assignment. just read and find out your weakness, your backlogs, try to cover it more • The scope of learning will increase	• It will be tough • Less practical experience • Less hands-on experience • Personal contact will disappear • Less social interaction with others • The education system may not change but to complete the academic year they may rush with left over syllabus and lectures and may affect the performance of the students and might not be at that satisfactory level

When we asked the participants “What will happen to neurorehabilitation post 2020?” Sixty percent of the participants responded to this question. A summary of responses is listed in Table 5.

**Table 5:** Views on neurorehabilitation post 2020.

**What will change?**	**Positive aspects**	**Suggestions**
• It will be different and we shall adapt • The only thing that I can think of is telerehabilitation • Done with great precautions to avoid any infection. Include more of home- based programs, easily carried out by relatives and actively by patient if possible, goal oriented programs. • Lot of work will have to be done due to increased cases of stroke post COVID-19 • Likely to incorporate more technology features • No difference post 2020 • The whole world is changing, and it is difficult to predict even for a day ahead. Hence, difficult to answer. • We may see a huge surge of post COVID-19 patients with neurologic sequelae. Our work may increase manifold. We need to be prepared. The frontline warriors today are fighting with the actual disease. • As recent research suggest probability of increase in cases of extra pyramidal involvement, this may be a challenge in neurorehabilitation	• Should become more advanced with patients becoming more aware • Good and advanced equipment should come into use for assistive technology • Good and explorative • With advanced knowledge many patients should be able to receive adequate treatment in holistic manner • More awareness • Neurorehabilitation will reach another destination holding the hands of telerehabilitation • Better understanding • More surge of disability expected as many will get delayed care at this juncture. So neurorehabilitation will need to gear up for that • Effective • Team approach would increase **Negative aspects** • Anxious about the changing techniques and quality reductions in improvements of neuro cases	• Standardization of scales • Qualified,accessible, affordable, and use of technology is the need of the hour in all forms of rehab • A lot of areas to be researched. • It is a field with a huge scope for growth. • It is also underrepresented. There is need for a unified national rehab council in India. • Need a lot of awareness and facilities at grassroots level • India should develop multidisciplinary rehabilitation unitsat each district in every corner of the country, uniformity of the services to all. • Dedicated Neuro-rehab Hospitals minimum 500 beds required to be made at the earliest. This is the need of the time. • De recognition of sub-standard colleges. • Multidisciplinary approach should be available for all the citizens of India at its best inclusive of rural, semi urban, and urban. • Policy makers and general public would become more aware toward especially needy persons

## Discussion

Neurological disorders remain a public health issue in India and the other developing countries. Neurorehabilitation is the main stay treatment to aid recovery and to minimize the morbidity in functional activities as a consequence of the disorder. There are various differences that exist in terms of neurorehabilitation service delivery in various parts of the country. Our study provides an overview of the influence of COVID-19 pandemic on the neurorehabilitation services and education in India.

Due to the surge in cases, the government and private institutes were converted to dedicated COVID care units, therefore inpatient neurorehabilitation admissions were reduced, emergency services were not available at certain centers for neurological diseases, and elective surgeries were canceled (5–8). However, certain centers were still taking care of emergency services as, early and prompt neurological interventions were needed to reduce the extent of injury and reduce the morbidity and mortality.

Post stroke rehabilitation is likely to be suboptimum during the pandemic and should focus on the most immediate needs of the patients. Telerehabilitation resources if available may be utilized (5). The pandemic made stroke care even more challenging. There is a need for public health systems in both developed and developing countries to improve awareness, implement proper strategies of triage, acute treatment, well-defined rehabilitation plans, telemedicine services, and virtual check-ins (6). Persons with Parkinson disease infected with COVID-19 are likely to have a motor and non-motor deterioration. As a result of social distancing, immobilization, and lockdowns necessitated by COVID-19, exercise, as well as physiotherapy or other rehabilitative services, maybe interrupted for PD patients. This lack of physical activity may lead to a worsening in the motor as well as non-motor symptoms (7). The longer the duration of ICU stay, the higher is the risk for long-term physical, cognitive, and emotional impairments needing comprehensive and early rehabilitation. We have to practically expand rehabilitation services in a resource-limited country, such as India, This would help to deal with the rapid increase in demand of post acute care facilities, be it in hospital services, in the form of inpatient or outpatient rehabilitation or home care facilities, including telemedicine (8).

Our research showed that majority of the rehabilitation units does not have a rehabilitation physician or a neurologist in the multidisciplinary team. One of the reasons for this can be discrepancy in the supply and demand of neurology services in our country as reported in the study by Khadilkar et al. (9) who found that among 1,800 neurologists in the country majority work in the metropolitan cities, thus, there is a scarcity of trained neurologists in rehabilitation services. Similarly, in a SWOT analysis, Shrivastava et al. reported that the lack of physiatrist in India due to education policies, as only a few medical colleges are providing post graduate training in physical medicine and rehabilitation, and lack of awareness of this new medical specialty among the medical fraternity, and the masses (10). Majority of the rehabilitation physicians are working in the metropolitan regions, leading to lack of adequate trained manpower in rural areas. Similarly, most of the members of the neurorehabilitation team including psychologist, speech and language pathologist, occupational therapist, and physical therapist also work in the urban areas. There is a substantial lack in the number of health care professionals for rehabilitation in low and middle income countries and frequently, the types of health care professionals needed for rehabilitation teams are not at all available. A few examples: high-income countries have, on average, more than 900 physiotherapists per million inhabitants; the corresponding number is <10 physiotherapists in many countries in Sub- Saharan Africa and the South-East Asia Region. Further, high income countries have more than 300 speech and language therapists per 1 million inhabitants, while some low-income countries in the African region have no speech and language therapists for the entire population. With this, there is thus a huge demand for education in neurorehabilitation. The need includes (a) the establishment of qualifying program for various disciplines in many countries, (b) specialized training in neurorehabilitation for health care professionals holding their basic professional qualification (physicians and allied health professionals), (c) continued medical education for those who have received specialized training, and (d) fast knowledge distribution in new challenging situations or “game-changing” opportunities for clinical practice (11). We suggest that proper training and appointments of neurorehabilitation professionals, and formation of specialized neurorehabilitation units especially in rural areas should be done to bridge this gap.

Countries across the globe have reported disruption of neurorehabilitation services during pandemic due to reduction in the number of beds for non-COVID patients and shifting of healthcare workers to COVID emergency duties (12–16).

There were different types of challenges in running neurorehabilitation services including clinical service challenge, health challenges for staff, clinical practice challenge, and capacity challenge. A sudden surge in admission of patients with a COVID-19 positive diagnosis has challenged all services, and has an impact on rehabilitation caseload management. Initially, the demand on the service increased mainly due to staff shortage, related to staff that had become infected with the virus, following government health instructions to self-isolate and stay at home until they were better (12). COVID-19 pandemic is strongly impacting all domains of our healthcare systems, including neurorehabilitation. In Italy, the first European country to be affected, medical activities were postponed to allow shifting of staff and facilities to intensive care, with neurorehabilitation limited to time-dependent diseases, including COVID-19 complications. Hospital access to people with chronic neurodegenerative conditions such as multiple sclerosis, movement disorders or dementia, more at risk of serious consequences from the infection, has been postponed (13).

COVID-19 has rapidly become a pandemic emergency, distressing health systems in each affected country. COVID-19 determines the need for healthcare in a large number of people in an extremely short time and, like a tsunami wave choking healthcare services. Rehabilitation services are also affected by this epidemic which forces radical changes both in the organization and in the operating methods (14). Unexpected rapid changes and reorganization of medical services that occurred during the pandemic lead to an impact in the practice of neurorehabilitation. The idiosyncrasies typical of neurorehabilitation management, especially in acute facilities that makes it susceptible as a vector of dissemination of COVID but also because of the need of finding new wards and intensive care units for COVID patients, the interventions in neurorehabilitation has suffered enormous changes (15). Our department is generally populated with a mixed age group of patients with numerous multiple comorbidities, which places them in a very risky situation. Immediate departmental recommendations have been put in place to safeguard these patients, including limitation of the number of visitors, higher thresholds for home visits and ward leave, limitations on social dining, and therapy sessions limited to the immediate bed space until newly admitted patients experience sufficient isolation (16).

A significant finding from our study has been how rural centers responded differently to pandemic than urban centers. Whereas, urban centers relied on telerehabilitation, rural centers were able to provide the outpatient services and follow up in more than 50% of respondents.

Due to the pandemic there has been an increase in the use of teleconsultation and telerehabilitation, which is feasible, easy, and cost effective to provide quality neurorehabilitation services (17). It is difficult to provide rehabilitation services to large numbers in public hospitals in the era of social distancing. Therefore, there is a need to change to newer and alternate mode of delivering the neurorehabilitation services like teleneurorehabilitation. The “new normal” has necessitated that practitioners and therapists quickly adapt to the changing needs of delivering rehabilitation care to patients. Findings from our study indicate that there has been uniform enthusiasm among rehabilitation professionals for use of telerehabilitation who have adapted to the technology well (18). This can be used in future to fill in the large gap between demand and supply on quality neurorehabilitation services in India (5–8). Telerehabilitation can be used for assessment, treatment, and follow up services, and even for educating the patients with neurological conditions. Telerehabilitation is beneficial for the patient psychologically as well, since the patient is rehabilitated in his home environment (19).

Remote communication technologies are increasingly regarded as potential effective options to support health care interventions, including neurorehabilitation and cognitive rehabilitation. Among them, telemedicine, virtual reality, augmented reality, and serious games could be in the forefront of these efforts (20). Growing evidence supports active video games and low-cost virtual reality as viable therapeutic interventions for children with physical disabilities. These technologies are especially well-accepted by pediatric populations for the ludic and motivating features that lend themselves to nearly seamless incorporation into telerehabilitation (21). Mobile Based Rehabilitation (MBR) offers many advantages: social distancing can be maintained by the indirect interaction/digital interaction of patient and therapist, easy and cost-effective method, reduces the travel costs and time consumption, convenient to access at any time, and entertaining method of rehabilitation, using games and virtual environments improves participation. Disadvantages of MBR includes difficult application for patients who have learning disabilities, cognitive impairment or psychological problems, access to the rural population due to poor resources, problems with network connectivity, and no manual contact with the therapists (22).

Our research has highlighted the effects of pandemic for the people with disabilities not only include physical effects such as increase in contractures and effect on therapy and treatment but also social effects like not being able to meet peers, family and friends, and participation restrictions, and psychological effects like frustration and depression. A list of these effects from open ended question will help the neurorehabilitation experts to plan comprehensive services for disasters and pandemics for the future. About 36% of the participants reported moderate to severe psychological impact, 25% showed mild to severe levels of anxiety, 41% reported depressive symptoms, and 41% felt stressed. Women, young, and those who lost their job during the health crisis showed the strongest negative psychological symptoms. We found factors associated with better mental health, such as being satisfied with the information received about the health crisis, conducting leisure activities, and the perception of being in good health (23). Early identification of distress and timely psychological interventions can, not only prevent crisis at times of pandemics but also help in containing its extent. The specific response to the mental distress of children who are quarantined should also be considered when designing psychological intervention strategies in response to COVID-19. Vigilance about the health of the elderly in long-term care is essential not only for their health but also to protect the health care system from being overwhelmed by severe COVID-19 cases (24).

There was an increase in participation for e-learning through webinars during the pandemic among the neurorehabilitation professionals. Carrying on this culture in the future to digitalize medical education, will be beneficial to the students and the staff as well, and it has been well accepted at present due to the current scenario (25). As reported in a few studies, e-learning has its own merits and demerits, it can be concluded that although traditional learning cannot be replaced by e-learning but adjusting and accepting the new normal is the way forward (26). Our research shows that education will change in the future and suggests that most accept a deviation to online teaching through web and VRbased systems. One of the findings from our study has been that responders have also looked at positive aspects of the change like Global access, Accessibility of education without barriers of geography and freedom to choose the subjects. Also responders feel that the change will stimulate new ideas and research. The concerns include adaptability, practical training, and distancing with less social interaction which is important part of education. The education policy makers will need to look at these aspects to minimize the effects. The boost which telerehabilitation has received due to the pandemic will go a long way in reducing the costs, treatment gap, and morbidities associated with neurological conditions

About the future of neurorehabilitation after 2020, our study indicated that it will be different and we will need to adapt. There will be surge in cases due to neurological consequences due to COVID-19 as well as sequel and complications due to lack of optimal care and therapy during pandemic. The suggestions for the future include that the neurorehabilitation should be accessible, affordable, and reachable to community and there is huge scope for improvements.

The results of our survey have lessons for the neurorehabilitation organizations, institutions, and professionals across the globe particularly developing countries. The study highlights the limitations in the reach of the neurorehabilitation services and effect of disasters such as COVID-19 pandemic on the delivery of neurorehabilitation services. It also highlights the potential use of technology in rehabilitation and education.

The limitations of the study include that it may represent views of 872 among 3,368 professionals contacted and may not present the true proportion of professionals in neurorehabilitation services. The questionnaire with multiple choice answers can influence the response process and have limitations of truthfulness and response bias. A questionnaire based survey also has limitations of over simplification of a complex reality and difficulty in determining the validity of data.

One of the unique features of our survey was inclusion of open ended questions. The answers to closed questions are influenced by the values chosen by investigators for each response category offered and that respondents may avoid extreme categories (27). Open ended questions allow unrestricted inquiry and lateral thinking, and should be included in a survey based research.

The survey has revealed positive aspects of the effect of COVID-19 pandemic in a developing country, and findings of the survey will be very useful for providing directions for future development of neurorehabilitation services, training, and education for all countries across the Globe.

## Conclusion

This study shows that there is paucity and imbalance of neurorehabilitation care in our country. There were social and psychological effects in addition to adverse physical effects to people needing neurorehabilitation during the pandemic. There was a difference in response of urban and rural centers to the pandemic where most of urban centers preferred telemedicine and stopped outpatient services, whereas more than 50% of rural centers continued outpatient services with precautions. Many professionals utilized the pandemic period to enhance their knowledge and skills through on line education such as webinars. The future of the neurorehabilitation is for a change if we utilize the learning from the pandemic period to make it accessible, affordable, and available. This study will guide health and education policy makers to design guidelines for neurorehabilitation training, continued education, and service delivery.

## Data Availability Statement

The raw data supporting the conclusions of this article will be made available by the authors, without undue reservation.

## Ethics Statement

Ethical review and approval was not required for the study on human participants in accordance with the local legislation and institutional requirements. The patients/participants provided their written informed consent to participate in this study.

## Author Contributions

NS developed the idea and guided the survey process. AS did the analysis and the write up process of the article. TN constructed the survey and analysis of the survey. DP helped in the process of development and conducting the survey. HS did the analysis of the survey and write up of the survey. All authors contributed to the article and approved the submitted version.

## Conflict of Interest

The authors declare that the research was conducted in the absence of any commercial or financial relationships that could be construed as a potential conflict of interest.
