# Supplementary material for: Education, Training, and Practices of Neurorehabilitation in India During the COVID-19 Pandemic
Source: Front Neurol. 2021 Feb 10;12:626399. doi: 10.3389/fneur.2021.626399 (PMC7902936; doi:10.3389/fneur.2021.626399)
Supplement: Supplementary file 1 [file Data_Sheet_1.PDF]

Education, Training & Practices of Neurorehabilitation in INDIA during the COVID 19 Pandemic

Greetings from Indian Federation of Neurorehabilitation.

Through this survey we aim to understand how Neurorehabilitation has been affected in times of COVID-19 in India & also what are the steps we can taken in the future to benefit the patients who require neurorehabilitation.

We request you to kindly fill the survey which will take 5 minutes of your time but will enable us serve the field of Neurorehabilitation in a better way.

If you have any queries or suggestions regarding this survey please contact ifnr2012@gmail.com

Thanking you for your active participation.
Kind Regards,
Dr. Nirmal surya Dr. Taral Nagda Dr. Abhishek Srivastava Dr. Deepak Palande
*Required

1. Email address *

____

2. Are you willing to Participate in this study? *

Mark only one oval.

( ) Yes *Skip to question 3*

Demographics

3. Age(Years) *

Mark only one oval.

- 20-24
- 25-29
- 30-34
- 35-39
- 40-44
- 45-49
- 50-54
- 55-59
- Above 60

4. Gender *

Mark only one oval.

- Female
- Male
- Other: ____________________

5. which city do you practice in? mumbai

____

6. Which state do you practice in? *

Mark only one oval.

- Andra Pradesh
- Arunachal Pradesh
- Assam
- Bihar
- Chhattisgarh
- Goa
- Gujarat
- Haryana
- Himachal Pradesh
- Jammu and Kashmir
- Jharkhand
- Karnataka
- Kerala
- Madya Pradesh
- Maharashtra
- Manipur
- Meghalaya
- Mizoram
- Nagaland
- Orissa
- Punjab
- Rajasthan
- Sikkim
- Tamil Nadu
- Telagana
- Tripura
- Uttaranchal
- Uttar Pradesh
- West Bengal
- Andaman and Nicobar Islands

- [ ] [illegible]
- [ ] Chandigarh
- [ ] Dadar and Nagar Haveli
- [ ] Daman and Diu
- [ ] Delhi
- [ ] Lakshadeep
- [ ] Pondicherry

7. Which Setup do you practice in ? *

Mark only one oval.

- [ ] Rural
- [ ] Semi Urban
- [ ] Urban

8. Specialty *

Mark only one oval.

- PMR
- Neurologist
- Neurosurgeon
- Orthopaedic surgeon
- Psychiatrist
- Orthotic and prosthetic Engineer
- Physiotherapist
- Paediatrician
- Cognitive therapist
- Occupational therapist
- Psychologist
- Speech therapist
- Ophthalmologist
- ENT
- Gastroenterologist
- Urosurgeon
- Social worker
- Special Educator
- Research Scientist
- Rehabilitaiton Nurse
- Others

9. Years of Experience in Neurorehabilitation *

Mark only one oval.

- Undergraduate Student
- Postgraduate Student
- PhD Student
- DM Neurology Resident
- Fresher
- 1-5 years
- 6-10 years
- 11-15 years
- 16-20 years
- 21-25 years
- More than 25 years

10. What Kind of Practice does your institute follow? *

Mark only one oval.

- Traditional Rehabilitation (Independent referral basis)
- Multidisciplinary Rehabilitation (Team Basis)

11. Your institute which you are affiliated to falls in which category? *

Mark only one oval.

- Medical college or teaching institute
- Private Hospital / Corporate Hospital
- Government Hospital
- Private Clinic
- Specialised Rehabilitation units
- Other: ____________________

Neuro-Rehabilitation Practice before COVID-19 Pandemic

12. Which Neurological conditions do you treat in your institute ? *

Tick all that apply.

- [ ] Amyotrophic lateral sclerosis (ALS)
- [ ] Alzheimer's disease & Dementia
- [ ] Aneurysm
- [ ] Bell's palsy
- [ ] Birth defects of the brain and spinal cord
- [ ] Brain injury
- [ ] Brain tumor
- [ ] Cerebral palsy
- [ ] Chronic fatigue syndrome
- [ ] Epilepsy
- [ ] Guillain-Barré syndrome
- [ ] Headaches and migraines
- [ ] Multiple sclerosis
- [ ] Muscular dystrophy
- [ ] Motor Neurone disease
- [ ] Neuralgia
- [ ] Neuropathy
- [ ] Neuromuscular and related diseases
- [ ] Parkinson's disease
- [ ] Psychiatric conditions (severe depression, obsessive-compulsive disorder)
- [ ] Spinal cord injury, Spinal deformity, Spine Tumors and disorders
- [ ] Stroke
- [ ] Vestibular disorders

Other: [ ] ______________________

13. How would you rate your Rehabilitation Unit *

Mark only one oval.

	1	2	3	4	5	
Basic	◯	◯	◯	◯	◯	Advance

14. 1How many Patients does your institute see on an average per month at an average? (Last 6 Months Statistics)-100 *

15. Does your Rehab Setup have the following- *

Mark only one oval per row.

	Yes	No	Not Applicable
PMR			
Neurologist			
Neurosurgeon			
Orthopaedic surgeon			
Psychiatrist			
Orthotic and prosthetic Engineer			
Physiotherapist			
Cognitive therapist			
Occupational therapist			
Psychologist			
Speech therapist			
Ophthalmologist			
ENT			
Gastroenterologist			
Urosurgeon			
Social Worker			
Rehab Nurse			
Music Therapist			

16. Which of the following rehabilitation modalities are available in your set up? *

Tick all that apply.

- [ ] Mirror Therapy
- [ ] Conventional Therapy (Tilt table, Parallel bars, Suspension therapy)
- [ ] Functional elecrical stimulation
- [ ] Upper limb Robotics
- [ ] Lower Limb Robotics
- [ ] Simple Treadmill
- [ ] Body Weight support treadmill
- [ ] Virtual Reality
- [ ] Repeated transmagnetic Stimulation
- [ ] Transcranial Direct stimulation
- [ ] Balance Master
- [ ] Vestibular rehabilitation
- [ ] Videofluroscopy
- [ ] Teleneurorehabilitation
- [ ] Acquatic therapy
- [ ] EMG Biofeedback
- [ ] EEG Biofeedback

Other: [ ] ____________________

Neurorehabilitation During COVID-19 Pandemic

17. During the LOCKDOWN due to the COVID-19 Pandemic, what services are being provided at your centre? *

Tick all that apply.

- [ ] Elective:
- [ ] Emergencies:
- [ ] New Patient OPD:
- [ ] Follow up OPD:
- [ ] Telerehabilitation

18. How often do you visit your work place *

Mark only one oval.

- Daily (Half day)
- Three times a week
- Twice a week
- Once a week
- Staying home

19. How are the patients continuing their rehabilitation *

Mark only one oval per row.

	No therapy	1%-25%	25%-50%	50%-75%	75%-100%
Independent					
Family based rehabilitation					
Private Caregiver based rehabilitation					
Therapist based rehabilitation					
Telerehabilitaiton					

20. What are the Protective Measures do you take while treating patients *

Tick all that apply.

- [ ] N95 Mask
- [ ] Surgical mask
- [ ] Gloves
- [ ] Hand disinfection
- [ ] Full PPE
- [ ] None

21. adapt to current situation and give services through Tele rehabWhat measures do you think should be taken by rehabilitation specialists to continue support to people needing rehabilitation *

22. What are three worst effects of Covid-19 pandemic for people with disabilities they are neglected more and where evr family are involved in rehab they are the one doing well others are deteriorated

Continuing Medical Education in times of COVID 19 Pandemic

23. How have you been utilizing the time available during pandemic *

Tick all that apply.

- [ ] Attending webinars on Neurorehabilitation topics
- [ ] Leisure
- [ ] Research
- [ ] Reading
- [ ] Hobby

Other: [ ] ______________________

24. What are the new skills you have learnt during the lockdown incontext to Neurorehabilitation?

25. How many webinars do you attend per week on an average ? *

Mark only one oval.

- less than 5
- 5-10
- 11-15
- more than 15

26. What format of webinars you feel are most effective during this lockdown? *

Mark only one oval.

- Lectures on Recent advances
- Panel discussion
- Case discussion
- Other: ______________

Opinon on Neurorehabilitation

27. Do you think Neurorehabilitation education is adequate in your fields curriculum in undergraduate and postgraduate courses? *

Mark only one oval.

- Yes
- No
- Maybe

28. Would you like to receive more training in the field of Neurorehabilitation? *

Mark only one oval.

- Yes
- No
- Maybe

29. Would you be interested in enrolling for any training courses organised by IFNR & WFNR *

Mark only one oval per row.

	Yes	No	Maybe
1 week course			
1 Month Course			
1 Year Course (Fellowship programme)			

30. Would you and your institute collaborate with any national society for multi-center studies? *

Mark only one oval.

- Yes
- No
- Maybe

31. How will education change in future after COVID 19 the online training will be an integrated part of rducation

32. many will be virtual onlineHow will conferences change in future after covid-19

33. What are your views on Neurorehab post 2020 it will be different and we shall adapt

This content is neither created nor endorsed by Google.

Google Forms
